# A Retrospective Study From a Single Center in Romania of 36 Patients Aged Between 37 and 59 Years Who Presented With Locally Advanced Colorectal Cancer

**DOI:** 10.7759/cureus.27572

**Published:** 2022-08-01

**Authors:** Anca Zgura

**Affiliations:** 1 Oncology, Carol Davila University of Medicine and Pharmacy (UMFCD), Bucharest, ROU

**Keywords:** colorectal cancer, young adult mean, recurence, gene mutations, risk factors

## Abstract

Background

The incidence of colorectal carcinoma is increasing in younger adults. This retrospective study was conducted at a single center in Romania and included 36 patients aged between 37 and 59 years who presented with locally advanced colorectal cancer. The purpose of this study is to show the importance of colorectal cancer screening in young patients.

Materials and methods

The study included 36 patients with histologically proven colorectal cancer evaluated in OncoFort Hospital. Disease staging was based on surgical findings and pre or post-operative abdominal CT or MRI of the abdomen and pelvis. The inclusion criteria were defined as a history of adjuvant chemotherapy plus radiotherapy and whether one had locally advanced colorectal cancer or recurrent or metastatic disease.

Results

Of the 36 patients, 13 (36.11%) were women, and 23 (63.8%) were men. The mean age was 47.4 years (range: 37-59 years). The colon cancers were more frequent than tumours of the rectum (n = 19, 52.77% versus n = 17, 47.23%). A total of 44.44% of patients were classified as stage III-IV. We found no significant correlation between mutation status or histologic grade and age.

Conclusion

This real-world study from a single center in Romania highlights that colorectal carcinoma may present in advanced stages in younger patients and may support consideration of a need to perform further studies to determine if the current age recommendations for screening should be lowered.

## Introduction

Colorectal cancer represents a significant public health problem worldwide, associated with high morbidity and mortality rates and a significant negative impact on patient's quality of life [1]. From an epidemiological point of view, this is the most common cancer of the GI tract, with global variations depending on the impact of various demographic and genetic risk factors [1].

During the last decades, the prognosis of patients diagnosed with colorectal cancer has improved due to the development of more effective systemic treatment options and the implementation of screening tests on a large scale [2]. By promoting the use of different screening methods such as colonoscopy, virtual colonoscopy, and fecal blood tests from a younger age in specific population groups at higher risk of colon cancer, the survival rates can be improved in correlation to early-stage diagnosis [3]. On the other hand, the research on molecular pathways and target therapies has increased the therapeutic response to chemotherapy [4-7].

The most important environmental factors linked to colorectal carcinogenesis are related to unhealthy dietary habits, smoking, and alcohol abuse. Over the past years, the fact that more and more cases of colorectal cancer have been diagnosed at younger ages can be associated with carcinogens found in processed food, such as high-fat and low-fiber diets, and also with low intake levels of calcium, selenium, and vitamins necessary for DNA repair and syntheses like folate and vitamin B12 [8]. Also, an unstable schedule of meals during the day or fasting regimens in young people can influence the body's metabolism and bowel movement, contributing to cellular alterations. Therefore, screening guideline changes to initiate younger colorectal cancer individuals without a family history or known risk factors should be considered for larger multicenter and population-based samples with additional well-designed research [9].

Colorectal cancer treatment is based on a multidisciplinary approach, including surgical options, chemotherapy, and radiotherapy [10]. Surgery is fundamental in treating colon cancer and may vary from an endoscopic resection of a malignant polyp to resections of large portions of the colon. Chemotherapy has shown a strong efficiency in limiting disease extension as a neoadjuvant treatment option and has a vital role in preventing local or distant recurrence of colorectal cancer after surgery [11]. Finally, radiotherapy is indicated in treating rectal cancer both in an adjuvant setting to prevent local recurrence and as a neoadjuvant approach to help downsize large tumors, allowing the preservation of the anal sphincter during surgical procedures [12,13]. 

A large retrospective study evaluated the incidence of colorectal cancer in a database consisting of over 143 million patients aged 20-49 years and found a value of 0.13%, with an increase of 7.9% per year in the 20-29 years old group from 2004 to 2016 [14]. In this trial, mortality had no significant changes in the youngest of patients but decreased slowly in patients aged 30-39 and 40-49, up to 2.4% per year.

This retrospective study, conducted at a single center in Romania, included 36 patients aged between 37 and 59 years who presented with locally advanced colorectal cancer.

Our purpose is to report the cases of our inpatient experience with seeing patients under 55 years old presenting with advanced colon cancer who never had screening. Our experience suggests that additional research in Romania is necessary. Whether earlier screening for colon cancer may lead to detection and removal of polyps and/or lead to fewer patients < 55 years old presenting with advanced colon cancer remains to be determined.

## Materials and methods

We conducted a retrospective, observational, and monocentric study based on patients suffering from locally advanced or metastatic CRC who met the inclusion criteria and were treated in OncoFort Hospital (Bucharest, Romania).

The cohort included patients diagnosed and treated between 2017 and 2020, for whom the follow-up period extended until 2021. The patient's history and records were collected in agreement with complete respect for anonymity and data protection as per the Declaration of Helsinki and its amendments. The study protocol was approved on November 16, 2020, by the Research Ethics Committee OncoFort Hospital (Bucharest, Romania) (approval number: 521) by the Institutional Ethics Committee.

Enrolled cases were defined as young patients presenting a histologically confirmed locally advanced, recurrent, or metastatic CRC and who benefited from adjuvant chemotherapy and/or radiotherapy. In addition, clinical and pathological features, such as gender, age, and mutation status, along with surgical findings and pre- or post-operative imaging by CT or MRI of the abdomen and pelvis performed in OncoFort Hospital, were recorded.

The chemotherapy regimen was delivered to eligible patients by our clinic-specific protocol (which includes supportive medication for primary prophylaxis of possible toxicities) until disease progression or unacceptable toxicity. Radiotherapy regimens consisted of 45-50.4 Gy delivered in 25-28 fractions of 1,8 Gy, using volumetric modulated arc therapy (VMAT) technique and daily image guidance while respecting specific organs-at-risk tolerance to radiation.

During treatment administration, essential aspects concerning the degree of toxicity according to the European Society for Medical Oncology (ESMO) and National Comprehensive Cancer Network (NCCN) guidelines' common terminology criteria for adverse events were updated in rapport with the number of courses, the time from the beginning of the administration to the last dose, treatment delay, the proportion of dose adjustment for each chemotherapy agent, and also data on the antitumor response. We took into consideration and tested genes reported to be mutated significantly in CRC, associated with a known germline mutational role in CRC development. Patient follow-up was based on clinical examination every three weeks, imaging using CT scan and serum levels of carcinoembryonic antigen (CEA) every three months, per international guidelines.

Data analysis was performed using SPSS 26.0 software (IBM Corp. SPSS Inc., Chicago, IL) by determining descriptive statistics such as medians and their interquartile range, frequencies, and percentages in order to describe the characteristics of the study population. The main statistical objectives of the study were represented by progression-free survival (PFS) and overall survival (OS) values. The most important parameters that influenced oncological response, irrespective of the number of courses administered, were chemotherapy dose reduction and treatment delays.

## Results

The results of data analysis of patients included in our study are consistent with an increase in CRC incidence in young adults aged 30-49 in Romania, with higher incidence findings among subjects aged 40-50 years. The cohort of patients was selected following inclusion criteria specific to young adult age, advanced or metastatic CRC, and the presence of genetic mutations. A total of 36 patients with locally advanced colorectal cancer were included in this study. Patients and tumor characteristics are summarized in Table 1. A total of 13 (36.11%) were women, and 23 (63.8%) were men. The mean age was 47.4 years (range: 37-59 years). The colon cancers were the most frequent tumors diagnosed in our cohort (n = 19, 52.77%), compared to rectum (n = 17, 47.23%) (Table 1).

**Table 1:** Clinical, pathological, and molecular characteristics of the study population. BRAF: v-raf murine sarcoma viral oncogene homolog B1; MMR: Mismatch repair; RAS: Rat sarcoma virus.

Characteristics of enrolled patients (n=36)	Frequency
Age	
Mean	47.11
Median	50
Gender	
Male	23
Female	13
Tumor site	
Colon	19
Proximal colon	8
Distal colon and rectum	28
Rectum	17
Metastatic setting	
Metastatic	13
Non-metastatic	23
Tumor grade	
G1 (Well-differentiated)	10
G2 (Moderately differentiated)	19
G3 (Poorly differentiated)	7
Treatment	
Surgery plus adjuvant chemotherapy	12
Surgery plus adjuvant chemotherapy and radiotherapy	14
No surgery	10
Oncologic treatment	
Chemotherapy alone	5
Radiotherapy and chemotherapy	17
Radiotherpay and chemotherapy + cetuximab	5
Radiotherpay and chemotherapy + bevacizumab	9
MMR status	
Stable	11
Un-stable	0
Missing data	25
RAS status	
Mutated	7
Non-mutated or not available	29
BRAF status	
Mutated	0
Non-mutated	4
Missing data	32

**Figure 1:**
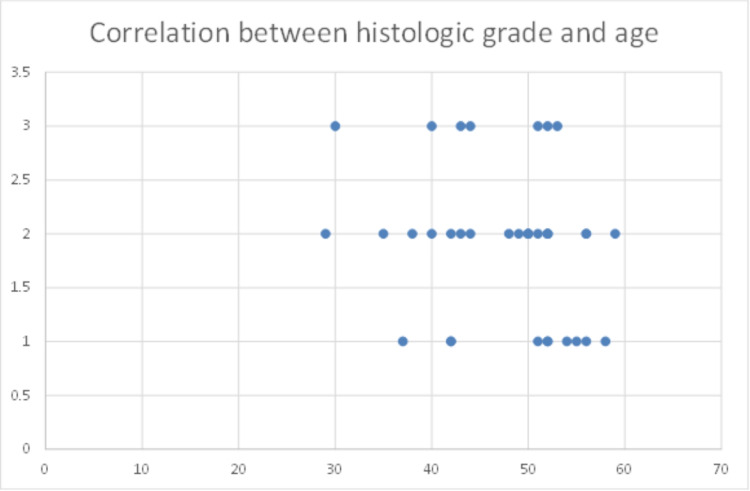
Association between histologic grade and age at colorectal cancer diagnosis.

The most common histological subtype of CRC was adenocarcinoma. Nineteen cases (52.77%) were moderately differentiated, ten cases (27.77%) were well, and seven (19.44) were poorly differentiated. Vascular invasion was seen in 16 cases (44.44%), was absent in 9 cases (25%), and was not determined in 10 cases (30.55%). Perineural invasion was identified in 13 (36.11%) tumors. In this study, the mean number of removed lymph nodes was 19.68 (range: 1-101). Positive lymph nodes (LNs) were identified in 12 (33.33%) patients. Regarding the pathologic stage, 10 cases (27.77%) of tumors were classified as stage I-II and 16 (44.44%) as stage III-IV. Metastatic disease (pathological stage) was diagnosed in three cases (8.33%), two patients with liver metastasis and one with peritoneal metastasis. We evaluated the presence of Kirsten rat sarcoma virus (KRAS), Neuroblastoma-RAS (NRAS), and v-RAF murine sarcoma viral oncogene homolog B1 (BRAF) mutations in the study group. In addition, we attempted a correlation between these mutations and the age and histologic grade. KRAS and NRAS mutations were detected in 19.44% (7 cases) of the examined tumors: 13.88% (5 cases) and 5.55% (2 cases) were found in KRAS and NRAS genes, respectively. In addition, BRAF mutations analysis was performed on four cases, none of which presented BRAF mutation (11.11%). We correlated the mutation status with histologic grade and age, and we found that there is a weak negative correlation without statistical significance. The tendency is for young patients to be associated with poorly differentiated histological points of view (Figure 1).

**Figure 2:**
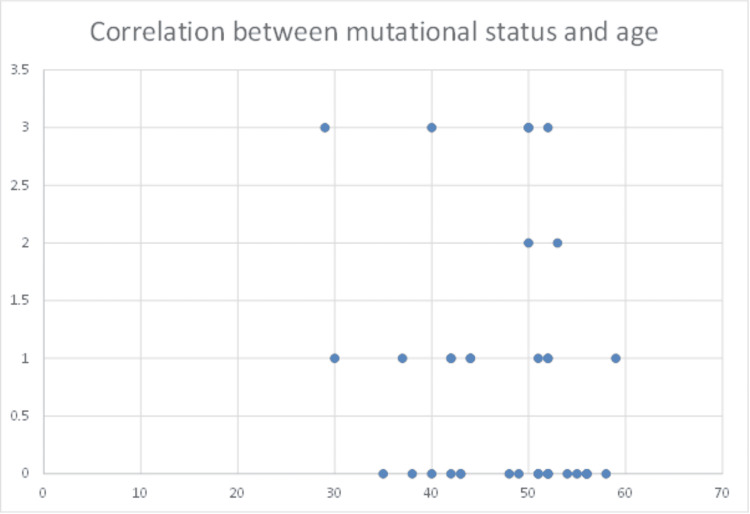
Association between mutation status and the age at colorectal cancer diagnosis.

We did not find a correlation between KRAS, NRAS, BRAF mutations, and age (Figure 2). It is known that the presence of mutations is correlated with the clinical course of CRC. It is interesting if it correlates with age too.

## Discussion

In our trial, we evaluated 36 patients aged between 37 and 59 years old diagnosed with locally advanced CRC. We found that most patients had moderately differentiated adenocarcinomas with frequent vascular invasion. The presence of KRAS, NRAS, and BRAF mutations did not correlate with age, but a weak negative correlation between age and histologic grading was observed. Compared to large trials [14], we evaluated patients of older ages, and unfortunately, mutational status was not determined in all cases. Nevertheless, the lack of association between the presence of specific mutations and age underlines the current opinion that screening for CRC should begin at ages much younger than previously recommended [14].

Due to the increasing global burden of CRC in terms of incidence and mortality rates, studies have been conducted in order to assess the correlations between young age at diagnosis as a risk factor and the prognosis. GLOBOCAN data from 2020 indicated that CRC represents the third most frequently diagnosed type of cancer and is associated with a high mortality rate, the second cause of cancer death [15]. Furthermore, the incidence of CRC at young ages (20-49 years) has increased during the past decades, according to reviews, from 9.3 per 100,000 in 1975 up to 13.7 per 100,000 in 2015 [16]. Therefore, a milestone in reducing the epidemiological impact of CRC is based on the promotion of primary prevention and early screening. According to the recommendations of the American Cancer Society, the age for screening initiation for individuals at average risk was lowered to 45 years in 2018 [17].

McKay A et al. [16] aimed to evaluate the influence of age at diagnosis on the survival rates of patients treated for CRC, whether with chemotherapy, radiation therapy, or surgery. The study is based on a retrospective analysis of a cohort of 2086 Canadian patients, 70 of whom (3.36%) were considered young (young age defined by the authors as less than 45 years). The study reviewed the outcome of patients diagnosed between 2004 and 2006, with a follow-up of five years. The group of patients had a median age of 72 years, with an age range varying between 20 and 103 years old. The results obtained by the authors regarding the prognosis for young patients (aged under 45 years) have shown better outcomes, with a 67.1% overall five-year survival, compared to patients aged between 45 and 80 years old (54.7%) and older than 80, respectively (33.4%). On the other hand, the subgroup of younger people was more likely to be diagnosed at a more advanced stage of the disease, with 22 patients diagnosed with stage III (31.43%) and 13 patients with stage IV CRC (18.57%) being more prone to present a T4 tumor (20 patients, 28.57% respectively) and positive LNs (N1: 19 patients, 27.14%, N2: 13 patients, 18.57%). Also, most patients (45, 64.29%) presented a G2 (moderate differentiated) adenocarcinoma. Overall, this study showed that even though most of the younger patients presented with more locally advanced disease, their prognosis was better than patients of an older age.

A retrospective study conducted by Chen FW et al. [17] compared data from two groups of patients diagnosed with CRC between 2008 and 2014, divided by age younger than 50 years: 253 patients, median age 43 (range 38-47), older than 50 years: 232 patients, median age 67 (range 59-75), p<0.001. The authors revealed that more young patients presented with advanced stage tumors (72%) compared to older patients (63%), (p=.03), also being more frequently associated with a family history of CRC (25% vs. 17% in older patients; p=0.03). Furthermore, 7% of the young group of patients had been diagnosed with hereditary cancer syndromes compared to older patients (1%, p<0.01). In the study's young group, the tumor's predominant site was the distal colon (41% vs. 34% in older patients; P=0.01) and the rectum (40% vs. 35% in older patients; P=0.29). Statistical findings concerning the cancer stage at diagnosis revealed that 71.5% of the young patients (181 cases) presented with advanced stages (stage III: 34%, stage IV: 37.5%), whereas 28.5% of the patients (72 cases) presented with stage I (7.9%) and stage II (20.6%), respectively. On the other hand, the older group of patients also presented more frequently with stages III or IV at diagnosis (145 cases, 62.5%) versus stages I and II (87 cases, 37.5%) (p=0.03).

Fu J et al. [18] also elaborated a retrospective study in order to evaluate the outcome of younger CRC patients. The data included in the study was based on a cohort of 2460 patients diagnosed with CRC between 1985 and 2011. The patients were divided into two groups, the cutoff age being 35 years old; thus, 140 cases (5.7%) were young patients, and 2320 cases (94.3%) represented the older group. Statistical results showed that more young patients were diagnosed with advanced cancer (stages III-IV), 69.3%, compared to the older group of patients (56.9%, p=0.000). Furthermore, the five-year OS rate in the younger group was 48.9%, while in the older group, the OS rate was 63.6%, concluding that the young patients had a worse prognosis and survival rate (p=0.046).

A study based on a prospective database comprised of 1327 patients that underwent surgery for CRC between 1990 and 2001 was published by Quah HM et al. [19]. The authors divided the cohort into two age groups, finding that 5% of the cases (68 patients) were diagnosed at a younger age (under 40 years old), while 95% of the patients (1259 cases) were more than 40 years old at the age of the diagnosis. As well as the other studies cited, younger patients were more likely to be diagnosed with cancers of the distal colon or the rectum (66% vs. 51%, p= 0.02). The predominant histological grade in younger patients was moderately differentiated adenocarcinoma (84% of the cases diagnosed in the group aged below 40 years old). All patients underwent complete surgical resection with clear histologic margins. The number of LNs removed in younger patients ranged between 11 and 30, with a median of 18, compared to older patients (9-21, median of 14) (p=0.001). Lymphovascular invasion was described in 12% of the younger patients and perineural invasion in 4% of the cases, with similar values obtained in the older cohort. As far as the stage at diagnosis is concerned, the cohorts studied by the authors did not present statistical differences regarding the distribution of tumor stage. Follow-up revealed that 18% of the younger patients (12 patients) and 17% of the older patients (211) developed local or distant recurrence. While the five-year recurrence-free survival was similar in both groups (79% in the young group versus 80% in older patients), the five-year OS rate was significantly higher in the younger patient cohort (84% vs. 73%, p=0.001).

A retrospective review by Al-Barrak J et al. based on the data collected from 16,732 CRC patients diagnosed between 1985 and 2005 found only 0.47% of cases (78 patients) to be diagnosed at a young age (considered by the author to be younger than or equal to 30 years old, but data was available for only 62 patients) [20]. Most of the cases diagnosed at a younger age had stage III cancer (49%), followed by stage II (42%) and stage I (9%). A larger number of patients had primary colon cancer (55% versus 36% in the rectum; 9% of the cases - 4 patients presented synchronous cancer). Most young patients had a well/moderately differentiated adenocarcinoma (76% - 34 cases), in opposition to poorly differentiated or anaplastic carcinoma (24% - 11 cases) [21]. Perineural and lymphovascular invasion was observed in 24% of the cases (11 patients). As a risk factor, a family history of CRC was observed in 38% of the cases. A total of 27% of the cases (17 patients) presented with metastatic disease, none of which reported positive family history of colorectal adenocarcinoma, but 29% (5 cases) presented inflammatory bowel disease. Most of the cases diagnosed with stage IV disease had a primary rectal tumor (7 patients - 41%). In comparison to patients diagnosed at stages I-III, a larger proportion of metastatic patients presented a poorly differentiated/anaplastic adenocarcinoma (47% - 8 patients versus 24% - 4 patients). From a statistical point of view, five-year overall survival was 44% in the entire cohort (54% in stage I-III and 12% in stage IV disease) [17].During the COVID-19 pandemic, early access to screening procedures and surgical interventions has been restricted due to the quarantine implemented globally as a measure of limiting the transmission of the virus. Also, the human and material resources of the healthcare system were redirected towards treating severe cases of COVID-19 [22]. Elective surgeries being postponed as temporary shutdowns were associated with a decrease in the number of patients that addressed their primary healthcare physicians for other pathologies than the infection with the SARS-COV2 virus. For the years to follow, this could translate into a higher incidence of locally advanced or metastatic cancers in patients who were not appropriately monitored since 2020 or whose surgeries were not performed in time, putting a strain on oncologists worldwide [23]. 

Our study has several limitations. First of all, this is a single-center retrospective study; therefore, we cannot extend our conclusions on a larger scale, taking into account the small number of patients analyzed. Second, a complete analysis of mutations was not available in all patients, which may have biased the statistical analysis. Also, due to the low number of patients, we could not divide young patients according to their respective age groups to perform pertinent comparisons to larger clinical trials.

## Conclusions

Early screening will bring benefits for adults beyond 40-55 years of age. Lowering the starting age of screening will have a favorable impact on the incidence and incidence-based mortality of the 40-55 age group. Therefore, monitoring CRC incidence in younger adults is essential to evaluate whether screening practices are effective. This real-world study from a single center in Romania highlights that CRC may present later in younger patients and supports the role of earlier national screening for CRC.
